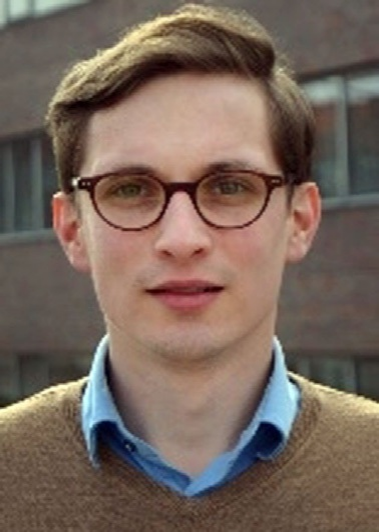# Direct Mechanocatalysis: Using Milling Balls as Catalysts

**DOI:** 10.1002/chem.202001177

**Published:** 2020-09-03

**Authors:** Wilm Pickhardt, Sven Grätz, Lars Borchardt

**Affiliations:** ^1^ Inorganic Chemistry I Ruhr-University Bochum Universitätsstraße 150 44801 Bochum Germany

**Keywords:** ball milling, cross coupling, heterogeneous catalysis, mechanochemistry, reactive milling

## Abstract

Direct mechanocatalysis describes catalytic reactions under the involvement of mechanical energy with the distinct feature of milling equipment itself being the catalyst. This novel type of catalysis features no solubility challenges of the catalysts nor the substrate and on top offering most facile way of separation.

## Graphical abstract

**High‐speed ball‐milling**: Direct mechanocatalysis is a new catalysis concept using high speed ball milling conditions. The surface of the milling balls is the catalytic active site, hence the recovery of the catalyst is convenient and efficient. Being a solid state technique, solvent related problems are omitted. Direct mechanocatalysis has proven itself as an environmentally friendly technique for various types of reactions, especially C−C coupling and polymerizations.

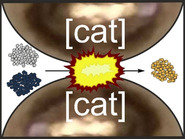

## Introduction

Catalysis is indispensable in chemistry and society. Most chemicals used in academia and industry have seen a catalyst at least once during their production. A big portion of the gross world product is related to goods that have required catalysis.^1^ Catalysis thus is rightfully one of the “Green Chemistry Principles” as it reduces the energy intensity of a reaction and prevents waste accumulation during synthesis.^2^ It is common to classify catalysis by the mean by which the activation barrier is overcome, that is, photons for photocatalysis, an electrical potential for electrocatalysis, or thermal energy for conventional thermal catalysis.^3^ A generally overlooked energy source, however, is mechanical energy, which may also initiate chemical and catalytic reactions.^4^ One way of transferring mechanical energy to the reactants is the collision of milling balls inside of ball mills.^5^ Reactions conducted this way are called mechanochemical reactions, if a catalyst is involved it is referred to as mechanocatalysis.^5^ This solid‐state technique has the great advantage of being solvent‐free. Moreover, it has widely been demonstrated that mechanochemical reactions can proceed faster, more energy‐ and resource‐efficient than conventional solution‐based reactions, thus making this discipline immanently sustainable.^1^ Additionally, frequently unexpected reaction pathways can be observed and sometimes even completely new products are accessible.^6^ All of these perks led to the classification of mechanochemistry as one of the ten emerging future technologies in chemistry by IUPAC in 2019.^7^

Catalytic reactions in ball mills range from C−C cross‐8 and homo‐coupling^9^ to Lewis acid and base chemistry^10^ and C−H activation.8a, 11 In all these examples, however, the catalyst is added as an additional powder, often simply adapted from the well‐known solution‐based reaction analogue or the reactions run autocatalytically.^12^ We denote these types of reactions as *indirect mechanocatalysis*.

In the special case that the milling equipment (e.g. the milling ball) itself is the catalyst, we want to introduce the term “direct mechanocatalysis” for this special concept. “Direct mechanocatalysis” is conceptually different in the following sense:

1. While homogeneous catalysis requires the catalyst and the reactants to be soluble in the same solvent, solubility and advanced ligand development becomes entirely obsolete for direct mechanocatalysis. It uses the crude catalyst, in many cases a ball made out of the required metal.
2. While heterogeneous catalysis converts fluid reactants (gases or liquids) on the desirably high surface of solid catalysts, direct mechanocatalysis preferably uses solid reactants and convert them on the smallest geometrically possible surface; a milling ball.
3. In the idealized conception, it is neither photons, electrical potential nor thermal energy that pushes the reaction above the activation barrier, but the mechanical energy of colliding milling items.

**Figure 1:**
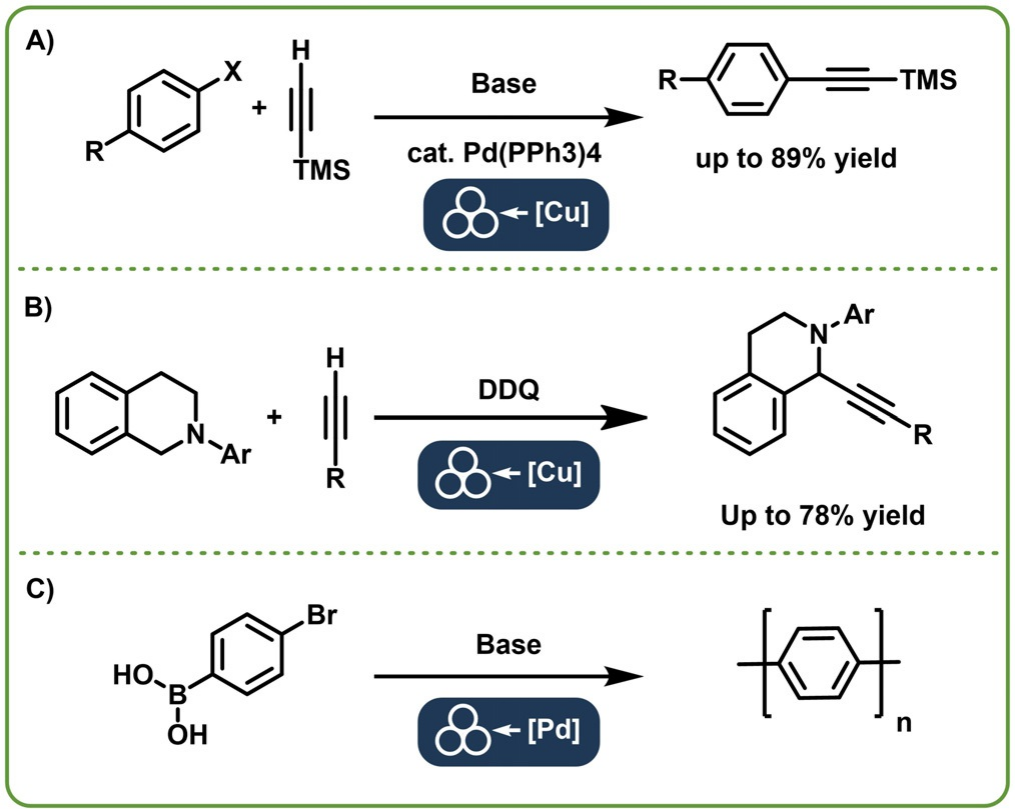
C−C coupling reactions using either palladium or copper balls or vials as catalyst species. We propose to combine the symbol for mechanochemical reactions introduced by Hanusa in addition with the catalytic element inside the milling balls as a symbol for direct mechanocatalysis. A: Sonogashira reaction with the replacement of the Copper co‐catalyst by the Mack group.^11^ B: Oxidative coupling catalyzed by copper balls by the Jiang group.8a C: Suzuki polymerization by Vogt et al.^9^

To dive into this topic, we want to exemplarily illustrate the first report on direct mechanocatalysis from 2009. Mack and co‐workers performed a mechanochemical Sonogashira reaction (Figure 1 A). In this pioneering work, palladium was still added as a catalyst powder but the co‐catalyst was Cu in the form of milling balls. This demonstrated that milling balls indeed participate in the reaction. They also conducted other reactions and consequently wrote a first mini‐review on this topic.^13^ Considering the concept of involving the milling equipment into a reaction, one may even go back as far as the first‐reported mechanochemical reaction at all. The famous reaction of cinnabar to elemental mercury in a copper mortar by Theophrastus of Eresos ca. 314 BC^14^ while not being catalytic, it already made use of the milling equipment during the reaction. On the following pages we want to introduce the concept of direct mechanocatalysis further, looking at: 1) *the reactions* by summarizing which reactions have already been performed via direct mechanocatalysis and are potentially viable; *2*) *the catalyst*, which is the milling ball, exhibiting a completely different composition and requirements than conventional catalysts; *3*) *the milling process*, including the role of the new reaction parameters that tend to be conceptually different than in solution‐based chemistry; 4) a discussion on *potential mechanisms*, a field still being in its infancy.

## The Reactions

At first, we want to present different reaction protocols using direct mechanocatalysis to introduce the scope of this technology. For now, examples are still sparse and only a small range of reaction types are represented in this field which will be summarized in this chapter. Later on, we will also discuss other potential reaction applicable to this principle.

### C−C coupling reactions

As the first reaction described to run via direct mechanocatalysis was the aforementioned Sonogashira reaction by the Mack group, we want to start this section with C−C coupling reactions. These reactions are commonly known for complex catalytic cycles, involving several reaction steps and often require co‐catalysts.^15^ At first glance, these reactions are thus not particularly suited for direct mechanocatalysis, since a simple ball or foil should hardly be capable to emulate the whole catalytic cycle. Surprisingly however, it was these reactions which were first investigated in this regard. Interestingly, the mechanochemical Sonogashira reaction was first established with the direct mechanocatalytic approach by the Mack group.^11^ Later also palladium salts were used in the absence of copper to achieve astonishing yields under solvent free conditions even without the inert atmosphere, which is commonly required in homogeneous Palladium catalysis.^16^

In their contribution the Mack group first develops a protocol for the solvent‐free Sonogashira reaction (Figure 1 A).^11^ During the screening of reaction parameters, they noticed that the addition of a copper iodide as co‐catalyst is not required for the mechanochemical reaction. However, by omitting the co‐catalyst the yield drops by more than 50 %. They then made the important step to replace the usual stainless steel or tungsten carbide milling ball with a copper ball bearing in the hope that the leaching of catalytic amounts of metal due to abrasion is enough to co‐catalyze the reaction. Since those experiments were successful, they went even further by producing their own copper milling vial. With this setup the yields were comparable with their first experiments, where copper iodide was used. They further stated that after each experiment, the ball and vessel were weighted and no significant change in weight, nor a depreciation of the yield over time was noticed. They therefore conclude that the co‐catalysis is indeed happening on the surface of the reaction vial.

The Jiang group, while working on cross‐dehydrogenative‐coupling reactions with alkynes (Figure 1 B), came across the results of the Mack group.8a They had been screening the addition of copper sources to enhance the reactivity and found that elemental copper led to good results. They also tried the use of copper balls and found that they could substitute the necessary copper, leading to yields rivalling those of copper salt‐catalyzed reactions. They further established that neither electron donating nor electron withdrawing groups had an influence on the yield under these reaction conditions, highlighting the advantages of the ball milling approach further.

The Suzuki cross‐coupling is another well‐established and investigated reaction in mechanochemistry.^17^ Inspired by these results, our group has recently shown that Suzuki cross‐coupling reactions, can be conducted via direct mechanocatalysis as well. While the Mack group replaced the co‐catalyst by active milling materials, we wanted to go one step further and completely eliminate the need for palladium salts in our reaction, since they are expensive and their reusability is limited. We chose a system on which we already established its benefits by transferring it from solution into the ball mill, the synthesis of poly(*p*‐phenylenes).^18^ In our recent work (Figure 1 C) we first demonstrated that no ligands or salts are needed and pure palladium black was capable of catalyzing the reaction inside a ball mill.^9^ In the next step we had palladium milling balls made and conducted the experiments inside a zirconium vessel with said balls. By studying the conditions further, we made the following observations: 1. the reaction is not proceeding in the absence of either palladium or base; 2. the reaction is reaching complete conversion slower under direct mechanocatalytic conditions compared to the palladium black or palladium salts. We, however, observed significant abrasion of palladium if the combination palladium balls and zirconia vessel was utilized. Softer vessel materials led to less abrasion without a reduction in yield. Therefore, we also reached the conclusion that the reaction itself has to happen on the palladium ball.

### Cycloaddition reactions

**Figure 2:**
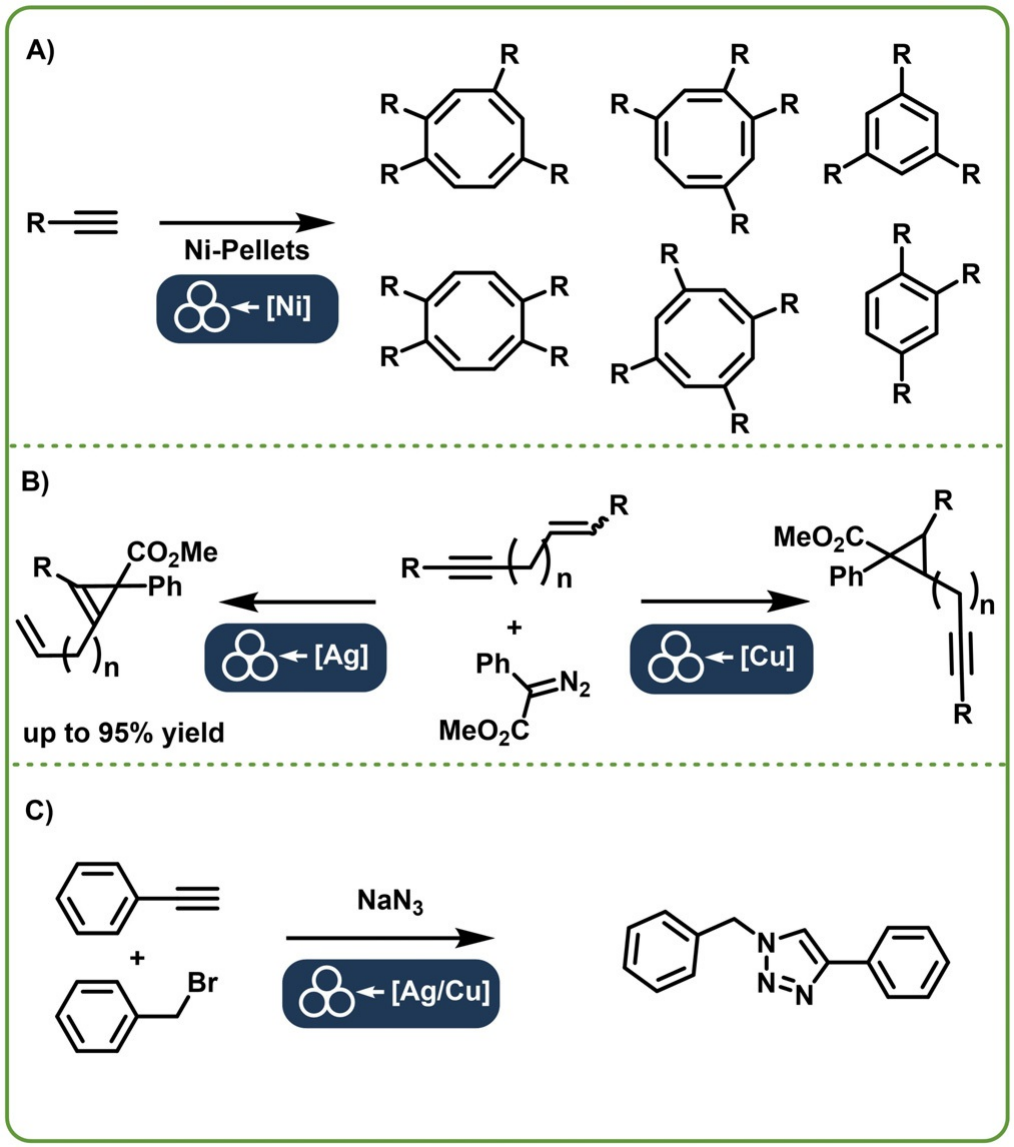
Cycloaddition reactions by Mack et al. A: Ni‐pellet catalyzed cycloaddition of alkyne derivatives leading to a mixture of cyclooctatetraene and benzene derivatives.^6^ B: Copper and silver‐catalyzed [2+1] cycloaddition. A competing reaction was set up to test the selectivity of the used metals.8b C: In situ generation and cycloaddition of an azide compound.8c

From 2016 onwards, the Mack group started to investigate cycloaddition reactions. While at first, they successfully utilized the known catalysts like Ni(PPh_3_)_4_, they quickly changed to the direct mechanocatalytic system. They started with a nickel‐catalyzed [2+2+2+2] cycloaddition reaction of an alkyne moiety (Figure 2 A).^6^ Since they could not find nickel balls they utilized pellets instead. The reaction lead to a mixture of various substituted cyclooctatetraenes and benzene derivatives, which was not expected according to solution‐based results, where benzene derivate dominate. During this investigation, the Mack group established that the product mixture is tunable by interchanging the substitution pattern of a phenyl acetylene substrate. This approach offered the use of an inexpensive, and recyclable Ni^0^ source and showed that direct mechanocatalysis can be conducted with readily available pellets instead of balls. Another, well investigated cycloaddition reaction was done by the Mack group as well. They established a coupling of a diazo compound to an unsaturated hydrocarbon (Figure 2 B). They expanded the concept to silver but instead of balls they used a silver‐lined vial.8b The established system was found to be reliable and robust in the synthesis of triazole derivatives.^19^ Here, the used metal foil had a direct influence on the obtained product. By switching between a copper or silver foil the position of the cyclization could be changed. The Mack group also applied the acquired knowledge to new reaction concepts. They developed a copper‐mediated generation and cycloaddition of organic azides (Figure 2 C) and successfully applied the direct mechanocatalysis of copper that they had observed earlier.8c The catalyst balls and foil‐lined vials allowed for both reactions to be performed in a one pot synthesis.

### Hydrogenation reactions

**Figure 3:**
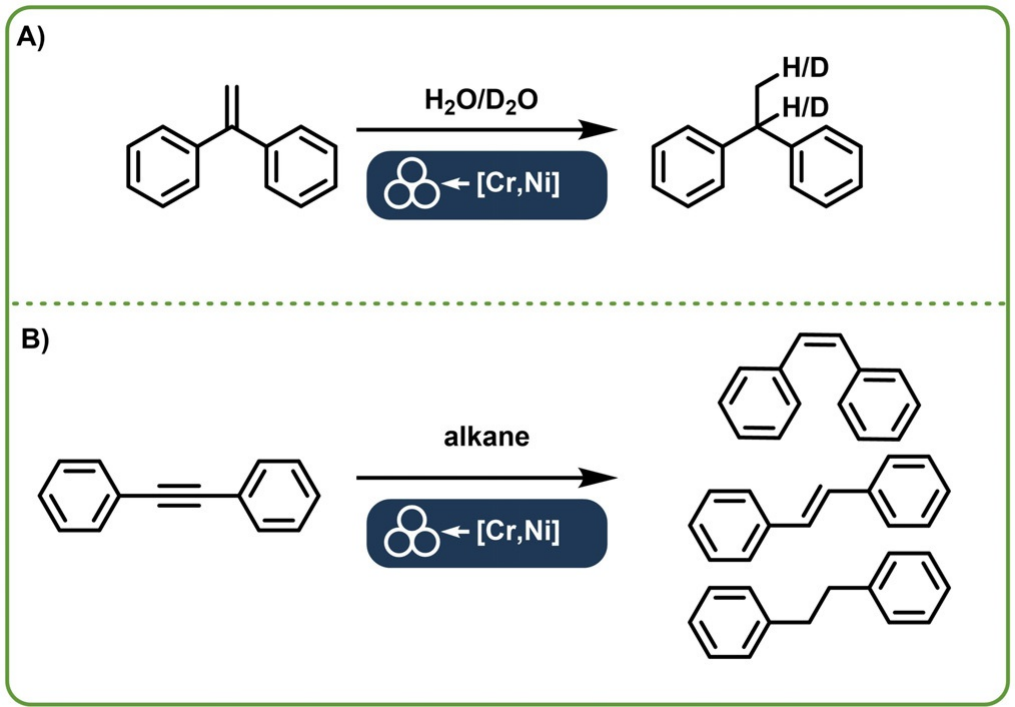
Dehydrogenation/hydrogenation reaction performed by the Sawama group. A: Water or deuterium oxide were dehydrated by the alloyed chromium. The in situ formed hydrogen was then used for a hydration of a substrate, utilizing the nickel, which is also present in the alloy.^17^ B: The described dehydrogenation/hydrogenation reaction using an alkane as hydrogen source.

A completely other type of reactions where direct mechanocatalysis has been successfully applied are hydrogenation reactions. While hydrogenation reactions themselves can be conducted via different methods inside the ball mill,^20^ the Sawama group found a unique reaction pathway using a stainless‐steel containing chromium and nickel. They were able to transfer hydrogen or deuterium from water or deuterium oxide, respectively to a substrate (Figure 3 A). During this transfer, chromium and nickel have distinct functions. Chromium is used to produce molecular hydrogen from water, while nickel is used to hydrogenate the organic compound.^21^ Further investigations of the same group showed how even organic molecules such as heptane can be used as a hydrogen source (Figure 3 B).

### Potential reactions

From these few literature‐known reactions we can already draw several conclusions on other reactions that might be accessible via direct mechanocatalysis. The activation of terminal alkynes, as well as copper‐catalyzed reactions are promising. Further, cross‐coupling reactions seem to be feasible as well, even if they are palladium‐catalyzed. Other reactions, which are in the focus of organic chemists are Ni^0^ catalyzed conversions. Due to the abundance of nickel, it would be a cheap and accessible catalyst, whereas the commonly used palladium is costly. Therefore, the application of nickel in the place of palladium in selective C−C coupling under mild conditions would also be promising. Going even further it might be feasible to conduct rhodium‐catalyzed metatheses reaction in the same manner.

It also stands to reason that this approach is not limited to organic synthesis alone. One should try to transfer common heterogeneously catalyzed reactions like MTO or even the Fischer–Tropsch process into a direct mechanocatalysis protocol. On the other hand, one is also not limited to molecular chemistry. As we have demonstrated this approach is feasible for the synthesis of polymers. It stands to reason that other polymerization reactions especially towards insoluble polymers can be conducted as well.

## The Catalyst

**Figure 4:**
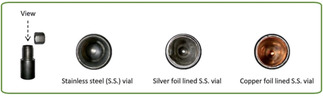
Milling vessels lined with copper and silver foil demonstrated by the Mack group.8b Published by The Royal Society of Chemistry.

After presenting the status quo, we want to discuss the reactions reported so far, regarding the requirements towards the applied catalyst. Besides the distinct catalytic activity, the catalyst has to be applicable in a milling environment. As the catalyst is the milling ball itself, it requires a certain breaking strength and hardness. Screening typical milling materials, this usually leads to ceramics and metals being applied. It is therefore not astonishing that particularly transition metals such as Ag, Cr, Cu, Ni and Pd have been in the focus of direct mechanocatalysis, demonstrating yields rivalling those from homogeneous reactions in solution.^13^, ^21^, ^22^ The aforementioned metals feature a common property of being quite soft and thus, not very resistant towards abrasion. In consequence, during the reaction fine metal powder is created, which might also act as catalyst. Therefore, the abrasion resistance is a challenge that needs to be tackled in order to elucidate mechanisms present in direct mechanocatalysis (Section 5). A potential method to circumvent the abrasion problem is to use alloys of the catalytic active metal. Metal alloys feature the advantage of being more resilient and can thus be used under milling conditions of higher energy. For several of the catalytically active materials there are harder and often cheaper alloys available (Table 1). To put the use of alloys into perspective: If we conduct a copper‐catalyzed reaction via direct mechanocatalysis and utilizes copper balls in a vibratory ball mill we often observe abrasion in the order of several hundred milligrams, or 10–20 % of the ball mass. If, however brass is utilized instead, the reaction proceeds faster and the abrasion is barley measurable. We thus postulate that one can substitute pure metals by alloys while keeping their catalytic activity comparable. In this context, the Sawama group recently published their results of a direct mechanocatalytic reaction.^21^ SUS304 was used as a catalyst material. This steel alloy consists mainly of three metals; iron, nickel and chromium. In their work they then proceeded to use pure metal powders with inert milling balls to elucidate the function of each of the metals present.^21^ This shows nicely, how a metal can be catalytically active under mechanochemical condition, even if the catalyst ball only contains 8–20 % of the catalytic active metal. Moreover, we want to highlight the possibility of conducting tandem reactions having two active metals within one alloy. The shape of the catalyst also plays a role. Usually round milling balls with polished surface are utilized in mechanochemistry. Different materials, however, are either not sold as balls or hard to shape into balls in the first place. For those cases it has been shown that even pellets and more rough geometries can be utilized as “milling balls”, as demonstrated nicely in the work of the Mack group on the nickel‐catalyzed [2+2+2+2] cycloaddition reaction (Figure 2 A).^6^ They went even further and utilized metal foils as a coating of the vessel to allow for a broad choice of milling items.^6^, ^13^ In those cases, the vessel and ball can then be chosen according to their density, hardness or abrasion resistance. While both approaches feature their unique advantages, their distinct disadvantages have to be considered. The Mack group used silver and nickel foil lined vessels (Figure 4) for two different conversions. The silver foil was used successfully in combination with a stainless‐steel ball without major losses of silver metal. In the case of the nickel foil, however, the best conversion was achieved when the nickel foil was used in combination with a tungsten carbide ball. This combination lead to a degradation of the foil, which was unusable after the reaction.^6^ Switching to nickel pellets leads to more impacts, but subsequently leads to microscopic particles, which are abraded during the reaction and need to be removed afterwards.^6^, 8b A commonly used compromise between possible number of impacts, abrasion resistance, availability and uncomplicated recovery is the use of 10 mm balls out of the desired material. The ball shape, albeit having the lowest surface to volume ration, features the decisive advantages of being easy to produce and featuring the highest resistance against abrasion.^6^, 8b, 9

**Table 1:** Potential catalytically active metal alloys and their components.

Metal	Nickel	Palladium	Copper
Alloy	Monel (NiCu33) Hastelloy (NiMo30) Nichrome (NiCr20)	PdCu30 PdAg40 PdSn15	Brass (CuZn35) Bronze (CuSn12) Aluminium Bronze CuAl10Fe5Ni5

Metal	Gold	Silver	Chromium
Alloy	Span Gold (AuCu18Al6) White Gold (AuNi10)	Platinum Sterling (AgPt5) AgCu25	Inox Steel Ferrochrome (FeCr50)

Metal	Iron	Platinum	Rhodium
Alloy	1.4301 (X5CrNi18‐10) 1.4104 (X14CrMoS17)	PtAu10 PtCu4	Pt30Rh ODS Pt20Rh FKS Pt10Rh

These observations are also supported by computational calculations. Here it has been shown that oblique collisions in a milling vessel produce a higher effective temperature than the impact of a ball and the vessel.^6^, ^14^ If this proves to be the case, a mechanocatalytic setup should be designed in a way that generates the most oblique collisions during the milling process.^6^, ^14^ Possible approaches to this challenge are the use of smaller grinding equipment or the use of foils made out of the catalytic active metal to increase the probability of an oblique collision.^6^, 8c, 13

## The Milling

After establishing the reactions and catalysts currently used in direct mechanocatalysis, it is important to take a look at the milling conditions needed for these types of reactions. A good starting point can focus on the types of mills that are commonly utilized. While there is a plethora of different mill types, only two of them are dominating mechanochemistry. These two types are the vibratory ball mills (also called mixer ball mills) and planetary ball mills. In vibratory ball mills the vessels are subjected to a horizontal, vertical or elliptical arc. Due to sudden changes in the direction the balls inside the vessel are colliding with the wall and each other. In this type of mill usually only a few balls (one to four) and rather simple and small milling jars are utilized. Together this makes for perfect systems for direct mechanocatalysis since even expensive materials can be manufactured into single balls and vessel geometries are simple and thus readily reproduced out of less common materials.

**Figure 5:**
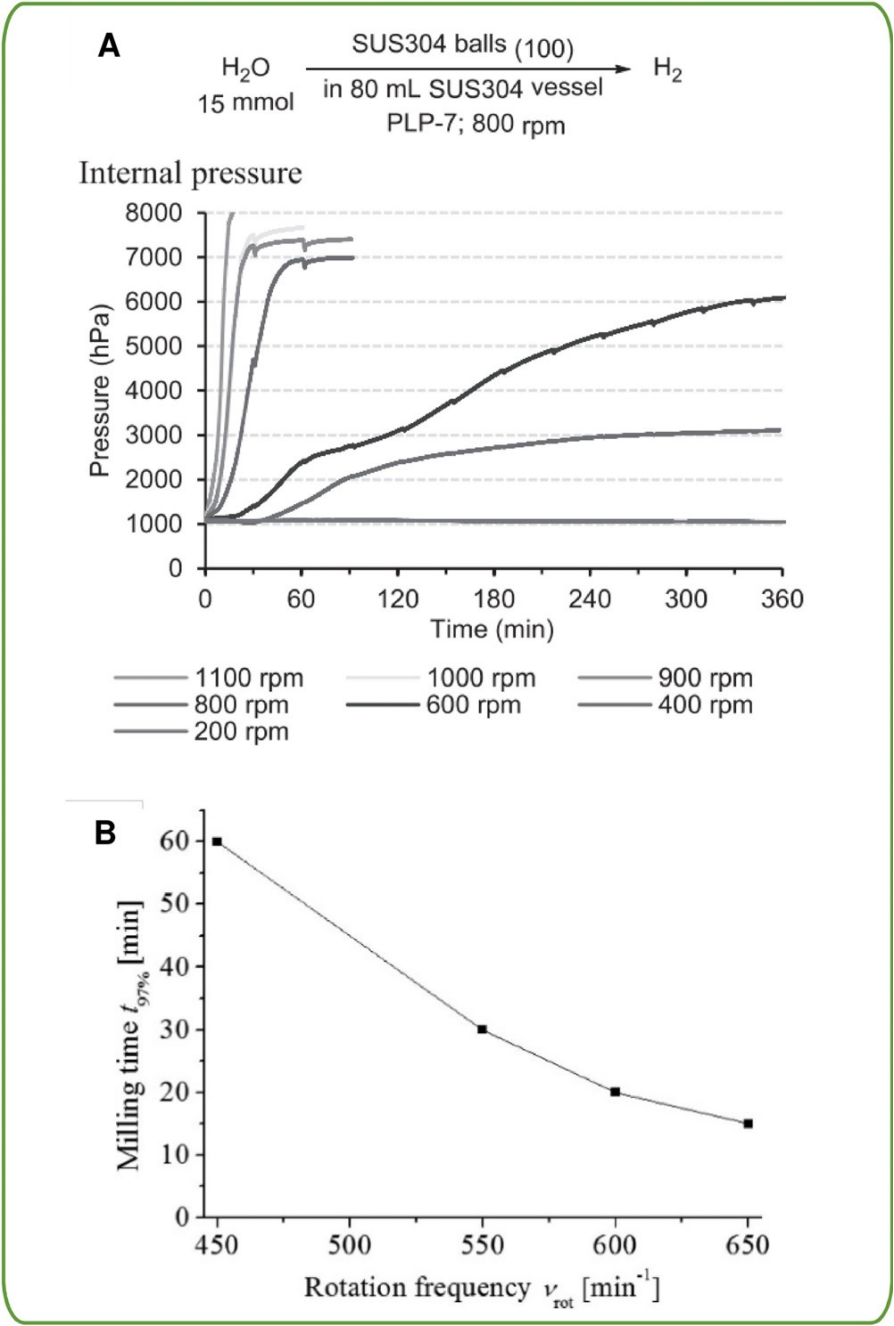
A: Influence of the rotational speed on the hydrogen evolution (pressure increase) from water in a planetary ball mill (P7). Copied from [17b] with permission of The Society of Synthetic Organic Chemistry, Japan. B: Influence of the rotational frequency on the time to reach 97 % yield of the Knoevenagel condensation of vanillin and barbituric acid in a planetary ball mil (P6) in a 250 mL steel beaker. Printed with permission from [19a]. Copyright 2015 American Chemical Society.

In planetary ball mills, vessels are moving on a sun wheel around an affixed point while rotating around their own axis simultaneously. These mills generally offer bigger reaction vessels with the downside of requiring more balls. However, for less expensive catalysts this milling geometry can also be adapted towards direct mechanocatalysis. Since no extensive studies on the importance of the mill type have been conducted for directly mechanocatalysis and the comparisons of different mill types has always been difficult for mechanochemical reactions, we can only extrapolate form the data points we have to our disposal. One difference between those two mill types is the ratio between sheer and impact forces during the milling. Here, the data indicates that the sheer forces, dominant in planetary ball mills, are more effective in conducting these types of reactions. Planetary ball mills often show shorter reaction times compared to mixer mills. This might be caused by the more frequent creation of new surface by the sheering motion as compared to the direct impact which rather compresses the powder present on the active surface.^23^ It might also be the case that the higher energy input in planetary ball mills is the sole reason for this apparent acceleration of reactions. The Sawama group could demonstrate that for their reactions higher rotational speeds are beneficial (Figure 5 A).21b

Besides the mill geometries there exist several easy to control and reasonably well‐understood parameters unique to mechanochemical reactions. As mentioned above, the rotational speed (planetary ball mill) or milling frequency (vibrational ball mill) are an easy to adjust parameter to control the energy input in the milled powder. In the past it has been shown, that by increasing the speed, the grain size of the obtained powder can be reduced and in general reactions are proceeding faster. (Figure 5 B) Additionally, the macroscopic temperature of the milling vessel is increasing since more energy is dissipated by heat. Therefore, it is hard to isolate the influence of the frequency and temperature terms in a mechanochemical reaction. Lately, Mack and co‐workers developed a cooled mixer mill setup with which they could demonstrate that the milling frequency alone can have an impact on the reaction.^24^ On the different end of the temperature spectrum the Group of Uzarevic has lately established a heatable milling setup. They observed drastic influences of moderate increases in temperature on the selectivity and reaction rate of mechanochemical reactions.^25^ For direct mechanocatalysis there is limited data on the impact of temperature and milling speed since most of the reaction have only been studied at one frequency in vibratory ball mills. In planetary ball mills, where temperatures are generally higher than in vibratory ones, Sawama established that at higher rotational speeds the catalytic release of hydrogen is faster. A closer look on their data suggest that especially in the low rpm‐range (400–800 rpm) this increase is mainly caused by the faster rotation since the temperature stays in the same range.21b At this point, however, more studies of the effect of temperature on those reactions, have to be done in order to obtain a conclusive answer.

The energy input can also be regulated by the density and size of the milling material. Since the milling material is of crucial importance in direct mechanocatalysis, this parameter is hard to change, except by using alloys. The ball size, however, is easier to alter. In general, small milling balls obtain a smaller kinetic energy at a given speed than their bigger counterparts. The minimal achievable grain size, however, gets smaller with smaller balls. Additionally, in neat grinding experiments, smaller balls tend to agglomerate if the reaction mixture melts during the milling process. Taking all of this into consideration typically milling balls between 5 mm and 15 mm are used, this stays true for direct mechanocatalysis.

Another important but seldomly investigated parameter is the milling ball filling degree—the amount of balls for a given vessel volume. Here, Kwade and co‐workers established that approximately 30 % of the vessel volume needs to be occupied by the balls for an optimal energy input. In direct mechanocatalysis this rule is generally followed in planetary ball mills.^6^, 21b, 26 The reactions in vibratory ball mills, however, usually only use one or two balls.^13^

Another interesting point is the combination/mixing of vessel and ball materials. It is often unwise to produce the full vessel out of a given catalytic material, be it due to cost or mechanical properties of the metal in question. In those cases, either a stainless steel or zirconia milling vessel is used with the required catalyst as ball, shot, or foil. If the differences in hardness, however, is too sever, the softer material is slowly being ground into powder by the harder one and massive abrasion has to be considered. This leads to immense wear^6^ and a drastic weight loss during reaction and contamination of the sample with metal powder. Even with only slight differences in hardness, this can produce a considerable amount of abrasion. In this case, it will be challenging to distinguish whether catalysis run on the surface of the balls or at the abraded metal powder. Again, alloys can help with this problem. For example, copper itself is a soft metal and not well‐suited for the use as milling balls, brass shows almost no abrasion while still catalyzing the copper‐catalyzed reactions.

## The Mechanism

The mechanism of mechanochemical reactions in general is poorly understood. This is caused by the unique reaction environment being a sealed and dense vessel that is rotating or vibrating rapidly. Conventional spectroscopic characterization setups established for solution‐based chemistry is hardly viable. Only in the last few years, in situ characterization setups have been developed that allow to shed light into the mechanochemical reactor. Namely, in situ X‐ray powder diffraction and in situ Raman spectroscopy have helped to identify reaction intermediates and propose mechanistic detail.^27^ These techniques are indispensable for direct mechanocatalysis, but probably not sufficient. The key question is: Where does the catalytic reaction run at? In an idealized view, it is the surface of the milling ball and the milling vessel. Although, abrasion during milling can be minimized due to wise selection of milling conditions, certain catalytic activity of the abraded species cannot be ruled out.^9^ In a recent publication, our group conducted a Pd‐milling ball‐catalyzed Suzuki reaction and exchanged the catalytically active milling balls against non‐active ZrO_2_ balls after 4 h of reaction. We observed that the rate of conversion decreased abruptly. That means that Pd abrasion accumulating during milling is not the major active specie in this reaction and the Pd milling ball as well as their collisions contribute to the overall reaction. We want to take this cross‐coupling reaction as a convenient example to discuss the challenge of mechanocatalytic reaction mechanism in more detail. Most cross‐coupling reactions known from solution follow reaction cycles involving oxidation addition and reductive elimination steps. Transition states are stabilized by solvents, catalyst ligands enable solubility and direct product selectivities. Neither a solvent, nor a ligand is present in direct mechanocatalysis, but the reaction proceeds anyway. In contrast, other conventionally used additives such as the base are still inevitable. Even more, many direct mechanocatalytic reactions appear to be highly sensitive to the type of base used. The HSAB concept seems to be of utmost importance as beautifully demonstrated by the group of James Mack.4b, 28 In this theory an atom, ion or molecule is defined either as hard (low polarizability, for example, F^−^) or soft (high polarizability, for example, Cs^+^). and a hard‐hard and soft‐soft pair is energetically beneficial.^29^ This can be visualized by the fact that LiOH (hard‐hard) is not capable of enabling the mechanochemical enolate reaction between 2‐methylcyclohexanone and bromobenzyl bromide, while NaOH (soft‐hard) is leading to a high yield.^28^ With this in mind, we strongly feel that direct mechanocatalytic reactions proceed differently to the well‐known solution‐based analogues. Reaction intermediates need to be identified and the catalyst itself needs to be investigated during reaction. This aims towards another fundamental question: Is direct mechanocatalysis a heterogeneous catalysis? From the concept it should be. Although all components can be solid, the milling ball is obviously a different phase than the reactants. This macroscopically sized catalyst can easily be removed after reaction. However, that the catalytic transformation runs at the surface of the milling ball has never been shown. Direct mechanocatalysis has certain features that are uncommon in established heterogeneous catalysis. Instead of fluid reactants (gases or liquids), solid reactants are preferably converted and instead of an ever‐higher catalyst surface, the lowest possible one of a dense macroscopic milling ball is chosen. Obviously, reaction rates are not solely determined by parameters such as temperature or the number of active sites, but by new parameters such as the frequency of active‐site‐refreshment caused by various milling parameters, for example, milling speed, density of milling balls, size of balls. Only very little is known about these types of reactions that is, catalysis on a surface under continuous mechanical impact. But how can we get a deeper insight into direct mechanocatalysis?

In the last years two in situ characterization methods have been well established by the mechanochemical community.^27^ The groups of Friscic, Uzarevic, Halasz and Emmerling in particular have done tremendous work on in situ powder X‐ray diffraction (pXRD).^30^ These techniques allowed for the tracking of reactions involving crystalline reagents in general and the identification of intermediates in specific cases.^31^ However, this technique is exclusively suitable for crystalline solids and long living intermediates since acquisition times of about 30 s are needed. Besides pXRD, in situ Raman spectroscopy has been applied for similar reactions.^32^ Here, no crystallinity is required but acquisitions times are still rather long (≈1–10 s) and the technique suffers from fluorescence. With a wise choice of laser parameters and reactants, however, it is possible to follow direct mechanocatalysis reaction with both in situ techniques.^33^ Both of these methods, however, can only give indications towards the mechanisms of direct mechanocatalytic reactions. To fully elucidate the mechanisms, one needs to closer investigate the milling ball surface in the moment of impact. This on the other hand seems hardly feasible and thus a proper investigation needs to heavily rely on ex‐situ data. One important step would be to identify intermediates or immobilized materials on the milling ball surface itself. Possible characterization methods could be TEM/SEM of a sliced of part of the milling ball, XPS of the milling ball surface before and after milling just to name two. As mentioned in the beginning, the field of direct mechanocatalysis is still in its infancy and besides first hunches no clear mechanisms have been established. It is the job of the mechanochemical community to dig deeper into this subject to uncover its full potential.

## Conclusions

We propose to use the term “direct mechanocatalysis” for catalytic reactions where the reactants are exposed to mechanical forces and the source of force is catalytically active itself. In general, the milling ball or vessel is made from a catalytically active component. This novel type of catalysis offers the advantage of directly using solid educts that do not require any solubility. Catalyst recycling and separation is most facile, as the macroscopic milling ball simply has to be taken out of the vessel after the reaction. Until now, this concept has been shown for only a few reactions, foremost transition metal‐catalyzed cross‐coupling reactions. However, if and how it can be adapted to other catalytic reactions will strongly depend on a better understanding of the underlying reaction mechanisms, which are expected to differ significantly from solution‐based reaction pathways. Since new reaction types and product selectivities are very likely by this approach, direct mechanocatalysis may not only enable new products and reactions, but also pave the way towards sustainable reaction alternatives.

## Conflict of interest

The authors declare no conflict of interest.

## Biographical Information

*Following his Master studies with focus on Organic Chemistry, Wilm started his PhD in 2019 at Ruhr‐Universität Bochum. He is working on direct mechanocatalysis, conducting the established liquid phase reactions under direct mechanochemical conditions*.

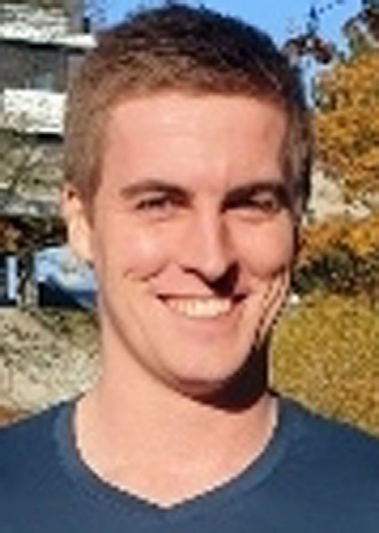

## Biographical Information

*Sven studied Chemistry and Chemical Engineering in Dresden and Strasbourg. He finished his PhD on the Mechanochemical Synthesis of Polymers in 2018 at Technische Universität Dresden. Currently he is senior scientist at Ruhr‐University Bochum working on mechanistic understanding of mechanochemical reactions as well as developing new synthesis concepts for porous polymers*.

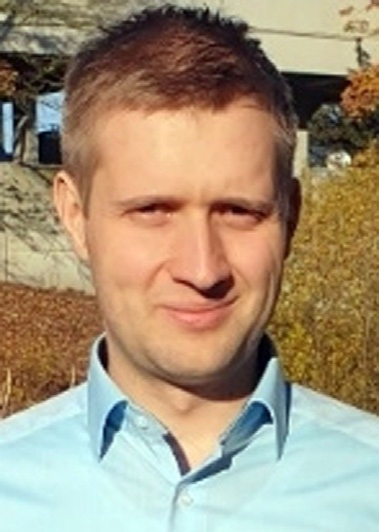

## Biographical Information

*Lars did his PhD in 2013 at Technische Universität Dresden in Inorganic Chemistry. After postdoctoral stay at ETH Zürich working on heterogeneous catalysis, he became leader of a junior research group working on mechanochemical synthesis of porous carbon materials for energy storage applications. In 2019 he was appointed professor at Ruhr‐University Bochum focusing on mechanochemistry entirely*.